# An RNA-sequencing-based transcriptome for a significantly prognostic novel driver signature identification in bladder urothelial carcinoma

**DOI:** 10.7717/peerj.9422

**Published:** 2020-07-21

**Authors:** Danqi Liu, Boting Zhou, Rangru Liu

**Affiliations:** 1 Department of Pharmacy, Xiangya Hospital, Central South University, Changsha, Hunan, People’s Republic of China; 2 Institute for Rational and Safe Medication Practices, National Clinical Research Center for Geriatric Disorders, Xiangya Hospital, Central South University, Changsha, Hunan, People’s Republic of China; 3 The Hunan Institute of Pharmacy Practice and Clinical Research, Xiangya Hospital, Central South University, Changsha, Hunan, People’s Republic of China; 4 Hainan Province Key Laboratory for Drug Preclinical Study of Pharmacology and Toxicology Research, Hainan Medical University, Haikou, Hainan, People’s Republic of China

**Keywords:** Bladder urothelial carcinoma, PCG-lncRNA-miRNA, Signature, Overall survival, Molecular subtype

## Abstract

Bladder cancer (BC) is the ninth most common malignancy worldwide. Bladder urothelial carcinoma (BLCA) constitutes more than 90% of bladder cancer (BC). The five-year survival rate is 5–70%, and patients with BLCA have a poor clinical outcome. The identification of novel clinical molecular markers in BLCA is still urgent to allow for predicting clinical outcomes. This study aimed to identify a novel signature integrating the three-dimension transcriptome of protein coding genes, long non-coding RNAs, microRNAs that is related to the overall survival of patients with BLCA, contributing to earlier prediction and effective treatment selection, as well as to the verification of the established model in the subtypes identified. Gene expression profiling and the clinical information of 400 patients diagnosed with BLCA were retrieved from The Cancer Genome Atlas (TCGA) database. A univariate Cox regression analysis, robust likelihood-based survival modelling analysis and random forests for survival regression and classification algorithms were used to identify the critical biomarkers. A multivariate Cox regression analysis was utilized to construct a risk score formula with a maximum area under the curve (AUC = 0.7669 in the training set). The significant signature could classify patients into high-risk and low-risk groups with significant differences in overall survival time. Similar results were confirmed in the test set (AUC = 0.645) and in the entire set (AUC = 0.710). The multivariate Cox regression analysis indicated that the five-RNA signature was an independent predictive factor for patients with BLCA. Non-negative matrix factorization and a similarity network fusion algorithm were applied for identifying three molecular subtypes. The signature could separate patients in every subtype into high- and low- groups with a distinct difference. Gene set variation analysis of protein-coding genes associated with the five prognostic RNAs demonstrated that the co-expressed protein-coding genes were involved in the pathways and biological process of tumourigenesis. The five-RNA signature could serve as to some degree a reliable independent signature for predicting outcome in patients with BLCA.

## Introduction

Bladder cancer (BC) represents one of the important common urological carcinomas and is the ninth most common malignancy worldwide (Siegel, Naishadham & Jemal, 2013). As a highly heterogeneous cancer, the development of BC is a multi-step process, and the majority of bladder tumours at present are low-grade non-invasive tumours (Siegel et al., 2014). The prognosis of BC patients remains poor, especially invasive BC. Stage progression was developed in some patients, meanwhile, about 30% of muscle-invasive BCs have occult distant metastasis at the time of diagnosis that led to a poor 5-year survival (Kamat et al., 2016). Bladder urothelial carcinoma (BLCA) accounts for 90% of bladder carcinomas. Due to the progression of these tumours and their frequent recurrence, the prognosis of BLCA patients is poor and their 5-year survival rate is only 5–70% (Siegel, Miller & Jemal, 2017). Although there have been advances in diagnostics and pre-operative and post-operative care, surgical techniques, chemotherapy and radiotherapy, there has been little distinct improvement in BC patients’ survival rates. As a result, the identification of novel clinical molecular markers in BC is still urgent to allow for predicting clinical outcomes. This study aimed at exploring the potential prognostic biomarkers for predicting survival in BLCA patients, which had much worse survival outcomes.

Over the past few decades, in response to the development of high-throughput sequencing technology, for instance the next generation sequencing (NGS), researchers have become devoted to uncovering novel molecular biomarkers from bulk sequencing data that have an impact on clinical outcomes by analysing the data at the transcriptome level or integrating multiple profiles with clinical data (Berger et al., 2018; Jiang et al., 2018; Liu et al., 2018b; Wang et al., 2018). Protein-coding genes (PCGs) are thought to be involved in many important pathways and biological processes during tumourigenesis (Ge et al., 2018; Liu et al., 2017; Zhang et al., 2017). A recent study found that Eukaryotic Elongation Factor-2 kinase (*eEF-2K*) expression was related to shorter overall survival in lung cancer patient (Bircan et al., 2018). A 24-gene hypoxia signature represented independent prognostic and predictive value for patients with muscle invasive bladder cancer (Yang et al., 2017). An 9-mRNA prognostic signature model (*EME1, AKAP9, ZNF91, PARD3, STAG2, ZFP36L2, METTL3, POLR3B, and MUC7*) has been found to be significantly associated with patients’ survival in muscle-invasive bladder (Han et al., 2019).

In recent years, long non-coding RNAs (lncRNAs) have been found to play an important role in many kinds of cancers involved in tumourigenesis and tumour progression. The differential expression analyses of lncRNAs in multi-cancer utilizing microarrays and RNA sequencing data have suggested that many lncRNAs are dysregulated in human cancers and many of these are related to patients’ prognoses (Yan et al., 2015). The upregulation of differently expressed lncRNAs (*PCAT-1* and *MALAT1*) is related to poor recurrence-free survival (RFS) of non-muscle-invasive bladder cancer (Zhan et al., 2018). Non-muscle-invasive BC patients with high *UBC1* expression had significantly lower recurrence-free survival (*p* = 0.01) (Zhang et al., 2019). The expression of lncRNA *HOTAIR* is a promising biomarker for predicting overall survival of patients with bladder transitional cell carcinoma (Shang et al., 2016). A prognostic 4-lncRNA (*AC005682.5*, *CTD-2231H16.1*, *CTB-92J24.2* and *RP11-727F15.13*) expression signature was established for BLCA and researchers believe that the signature has a good predictive ability (Bao, Zhang & Dong, 2017). Liu et al. (2018c) proposed a prognostic 5-lncRNA Expression signature for patients with head and neck squamous cell carcinoma. Fan, Ma & Liu, (2018) constructed a competing endogenous RNA (ceRNA) network with lncRNA-miRNA-mRNA and identified a four-lncRNA signature (*ADAMTS9-AS1*, *LINC00536*, *AL391421.1* and *LINC00491*) that could independently predict overall survival (OS) in breast cancer (BC) patients.

Apart from PCGs and lncRNAs, miRNAs are also considered to be dysregulated expression related to multiple cancers. Researchers have come to the conclusion that a five-miRNA signature (*hsa-let-7g-3p*, *hsa-miR-6508-5p*, *hsa-miR-210-5p*, *hsa-miR-4306* and *hsa-miR-7161-3p*) is a strong and independent prognostic factor in predicting disease recurrence and survival of patients with HPV-negative head and neck squamous cell carcinoma (HNSCC) (Hess et al., 2019). For these miRNAs, in addition to head and neck cancer (Gee et al., 2010; Hess et al., 2019), *hsa-mir-210* has already been reported to have an impact on pancreatic cancer (Greither et al., 2010), osteosarcoma (Cai et al., 2013), renal cancer (McCormick et al., 2013), non-small cell lung cancer (Eilertsen et al., 2014), glioblastoma (Qiu et al., 2013), soft-tissue sarcoma (Greither et al., 2012) and breast cancer (Buffa et al., 2011; Rothe et al., 2011; Volinia et al., 2012). Concerning bladder cancer, a nine-miRNAs (*hsa-miR-99a-5p*, *hsa-miR-100-5p*, *hsa-miR-125b-5p*, *hsa-miR-145-5p*, *hsa-miR-4324*, *hsa-miR-34b-5p*, *hsa-miR-29c-3p*, *hsa-miR-135a-3p*, and *hsa-miR-33b-3p*) provides prognostic and predictive value of patients with urothelial carcinoma of the bladder (Inamoto et al., 2018).

In summary, PCGs, lncRNAs and miRNAs are potential biomarkers predicting survival in tumour patients, the study of Robertson et al. (2017) only focused on a unilateral special transcriptome signature that has its limitations. In the case of BLCA in this study, we integrated PCGs, lncRNAs and miRNAs expression data from a large dataset (*n* = 400) in The Cancer Genome Atlas (TCGA) database to predict the overall survival of patients with BLCA more precisely and utilized non-negative matrix factorization (NMF) and similarity network fusion (SNF) to classify patients with BLCA into distinct molecular subtypes.

## Materials & Methods

### Data source and preprocessing

**Figure 1:**
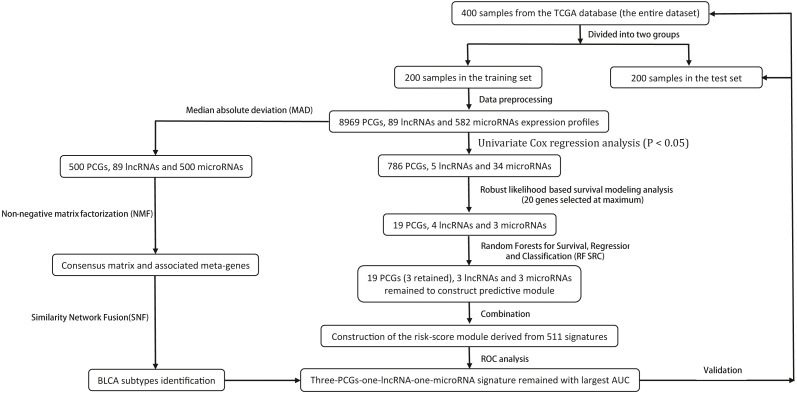
Process of the study. The procedure of analyses to construct a risk score model and evaluate the prognostic power of this obtained signature and the BLCA subtypes identification.

Gene expression profiles (Illumina HiSeq RNA Seq), miRNA mature strand expression profiles (Illumina HiSeq microRNA Seq) level 3 data and phenotype data of bladder urothelial carcinoma (BLCA) (Robertson et al., 2017) from the TCGA database were downloaded through the UCSC Xena portal (https://xena.ucsc.edu/). The mRNA expression profiles and lncRNA expression profiles were derived from the entire gene expression profiles. Genes with missing expression values in > 50% samples were removed. Patients chosen for model constructing met the following criteria: (1) histologic diagnosis of primary BC; and (2) available RNA expression profiles and complete clinic-pathological and follow-up data. After sample filtering, four hundred patients were enrolled for further analysis and divided into 200 samples in the training set and 200 samples in the test set randomly based on the “sample” algorithm. The “sample” algorithm was a basic function in R. The application was generating random samples. This means that sample takes a sample of the specified size from the elements of x using either with or without replacement. We utilized this function to guarantee the randomness of the samples and repeatability of results and avoided the sample-specific results. The selection process of the prognostic signature is shown in Fig. 1.

### Preliminary screening of generally changed RNAs

First, RNAs with significantly high variability expression among different patients of primary bladder cancer were preliminary screened. Because these RNAs could be considered as important roles in tumorigenesis. A generally changed gene was selected based on the criteria outlined below:

1. The median expression of gene in every sample was more than 20% of the total median expressions of all RNAs in every sample;

2. The variance of expression of gene in every sample was higher than 20% of the total expressions variances of all RNAs in every sample.

### Selection of seed RNAs

All of the selected RNAs with significant changes across patient samples were analysed by univariate Cox regression survival analysis to evaluate the relationship between the expression of each PCG, lncRNA or microRNA and patients’ OS by “survival” package in R in the training set (Grambsch & Therneau, 2000; Therneau, 2015). RNAs found to have significant *p* values < 0.05 were selected as seed RNAs, indicating prognostic RNAs.

### Identification of key RNAs related to prognosis

Considering that a few PCGs, lncRNAs and microRNAs in one model would make the prediction more precise and practical, we selected the top 20 PCGs, lncRNAs, and microRNAs to perform the robust likelihood-based survival models to identify key biomarkers influencing the clinical outcome of cancer by utilizing the “rbsurv” package in R language (Hyung, Cho & Yu, 2009; Kendall, Pollock & Brownie, 1995; Renaud et al., 2015; Wang & Tai, 2009).

Furthermore, we performed the random forests for survival regression and classification (RF-SRC) algorithm by using the “randomForestSRC” package in R language to filter genes until three PCGs, three lncRNAs and three microRNAs were retained to construct a predictive model (Ishwaran & Kogalur, 2010; Ishwaran et al., 2014).

### Screening of BLCA subtypes-associated metagenes

Through data preprocessing, we screened out 8969 PCGs, 89 lncRNAs and 582 microRNAs. Before performing NMF, we applied a filtering procedure to retain genes with high variability across 200 patients from the training cohorts because the higher variable genes are informative in the clustering process (Zhao, Zhao & Yan, 2018). The median absolute deviation (MAD) value of each gene was calculated, and we selected the top 500 most variable genes for the clustering analysis. In particular, the 89 lncRNAs expression in the 200 patients were all reserved for the following analysis.

The “NMF” R package (Gaujoux & Seoighe, 2010) was utilized to perform the clustering analysis with the Brunet algorithm. Informative genes were extracted by NMF method. We used NMF to reduce the dimensionality of expression data from thousands of genes to a handful of metagenes (Brunet et al., 2004). The number of clusters k was varied from 2 to 10, and we repeated the clustering process 30 times. The value of k that led to the stable cophenetic correlation coefficient mentioned by Brunet et al. was chosen as the optimal number of clusters. Next, we repeated the clustering 200 times with an optimal k to obtain the associated metagenes and consensus matrix. Subtype-specific genes were singled out using the “extractFeatures” function with the largest row feature scores in the “NMF” package (Gaujoux & Seoighe, 2010).

### Identification of BLCA subtypes

We clustered the 200 tumour samples in the training set using the SNF algorithm based on the “CancerSubtypes” R package (Xu et al., 2017). Then, we selected 20 as the number of iterations for the diffusion process. The statistical significance of the clustering (SigClust) was calculated to validate the significance of the clustering results. This calculation assesses the significance of clustering by simulation from a single null Gaussian distribution. The parameter of the “icovest” was set as 3, indicating original background noise threshold estimate. Survival analysis was also conducted to validate the significance and verify the survival patterns between the identified molecular subtypes or in every cancer subtype. We also made a comparison between our classification and the existing subtype classification from the TCGA subtypes (Robertson et al., 2017). The heatmap corresponding to the dendrogram was generated using the heatmap function with SNF algorithm classification, mRNA cluster, lncRNA cluster, microRNA cluster, RPPA cluster, hypermethylation cluster, hypomethylation cluster, mutation process (MSig) cluster, SMG-SCNA cluster, histological subtype, TNM stages, clinicopathological stages, histological grade, and TP53 mutation, KRAS mutation, BRAF mutation and EGFR mutation as the annotations.

### Multivariate survival analysis

To describe how these RNAs affected the prognosis of the BLCA patients, multivariate survival analysis was performed on the 511 signatures composed of the selected 9 biomarkers using permutation and combination method in the training dataset by using the “survival” package in the R language. Subsequently, we established a weighted overall survival (OS) prognostic index algorithm model for prediction of the prognosis using the following:

Risk score (RS) = ${\mathop{\sum }\nolimits }_{i=1}^{N} \left( Exp\mathrm{ \ast }Coef \right) $

where *N* was the number of prognostic PCGs, lncRNAs and microRNAs in the model, *Exp* stands for the expression value of the PCGs, lncRNAs and microRNAs, and *Coef* was the estimated regression coefficient of the PCGs, lncRNAs and microRNAs in the multivariate Cox regression model. Patients who have higher risk scores are expected to have a higher probability of a poor outcome. Selecting the median risk score in each dataset as a cutoff value, bladder urothelial carcinoma patients were divided into high- and low- risk groups (Zhou et al., 2015). Meanwhile, we performed Kaplan–Meier survival analyses to inspect the differential for survival distributions in different groups for each BLCA cohort, and the two-side log-rank test was used to assess the statistical significance. Furthermore, multivariate Cox regression analysis was conducted to test whether the risk score was independent of other clinical covariates.

### Functional annotation via GO, KEGG analyses and gene set variation analysis (GSVA)

To further investigate the underlying biological roles and pathways of the three-dimension transcriptome signature, the co-expression relationships of the three PCGs, one lncRNA and one miRNA with the corresponding co-expressed protein-coding genes were calculated using Pearson correlation coefficients in the training dataset. GO analyses were conducted by using R package clusterProfiler (Yu et al., 2012). GSVA (Hanzelmann, Castelo & Guinney, 2013) was applied to obtain the abovementioned co-expressed PCGs based on the following criterion: Pearson correlation coefficient > —0.3—, *p*-value < 0.05.

### Statistical analysis

Survival analysis was performed using the Kaplan–Meier method, and the differences between survival curves was assessed using the log-rank test. Univariate and multivariate analyses were conducted using Cox proportional hazard models. Survival predictive accuracy of prognostic models was estimated based on a receiver operating characteristic curve (ROC) analysis via “pROC” R package (Robin et al., 2011). All statistical analyses were performed in the R platform (R Core Team, 2019). All statistical tests were two-sided and *p*-values < 0.05 was considered statistically significant.

## Results

### BLCA patients’ characteristics in the training and test sets

A total of four hundred patients included in this study were pathologically diagnosed with bladder urothelial carcinoma after preprocessing. Patients with missing overall survival data were excluded from this study. These patients were divided into a training set (*n* = 200) and a test set (*n* = 200) randomly and evenly. The patient age was classified as ≤ 61 and according to the X-tile software (Yale University Version 3.6.1, http://tissuearray.org) (Camp, Dolled-Filhart & Rimm, 2004). All of the baseline demographic and clinical characteristics of these three datasets are summarized in Table 1.

**Table 1:** Summary of patient demographics and clinical characteristics of datasets.

**Characteristics**	**Training set(*n* = 200)**	**Test set(*n* = 200)**	**Entire set(*n* = 400)**
**Age, years**			
≤61	60	58	118
>61	140	142	282
**Gender**			
Male	149	147	296
Female	51	53	104
**Pathologic stage**			
Stage I	1	1	2
Stage II	58	70	128
Stage III	70	66	136
Stage IV	70	62	132
Unknown	1	1	2
**Histologic grade**			
High grade	189	188	377
Low grade	10	10	20
Unknown	1	2	3
**Pathologic T**			
T2	44	48	92
T3	97	92	189
T4	22	20	42
Unknown	37	40	77
**Pathologic N**			
N0	114	117	231
N1	24	21	45
N2	37	38	75
N3	4	3	7
NX	16	20	36
Unknown	5	1	6
**Pathologic M**			
M0	101	93	194
M1	7	4	11
MX	89	103	192
Unknown	3	0	3
**Diagnosis subtype**			
Papillary	63	64	127
Non-papillary	135	133	268
Unknown	2	3	5
**Overall survival (years)**	2.29 ± 2.41	2.17 ± 2.18	2.23 ± 2.29
**Vital status**			
Living	110	115	225
Dead	90	85	175

### Identification of significant PCGs, lncRNAs and microRNAs associated with overall survival from the training set

Through preprocessing and preliminary screening of the significantly changed PCGs, lncRNAs and miRNAs in the training set (see methods), 8969 PCGs, 89 lncRNAs and 582 microRNAs expression profiles were generated for key RNAs identification.

We utilized three algorithms to identify the central RNAs used to construct the candidate prognostic prediction models. First, we conducted a univariate Cox proportional hazards regression analysis with the PCGs, lncRNAs and microRNAs expression profiling data as the independent variables while survival time and survival status were the dependent variables, and we identified 785 PCGs, 5 lncRNAs and 34 microRNAs that were significantly associated with patients’ OS (Table S1). Second, we used the robust likelihood-based survival models to identify 19 PCGs, 4 lncRNAs and 3 microRNAs that were the most highly correlated with the prognostic information. Third, using the random forests for survival regression and classification algorithm, we finally screened out 3 PCGs, 3 lncRNAs and 3 microRNAs according to the variable importance (VIMP) (Fig. S1).

### Acquisition and construction of the prognostic PCG-lncRNA- microRNA signature model in the training set

**Figure 2:**
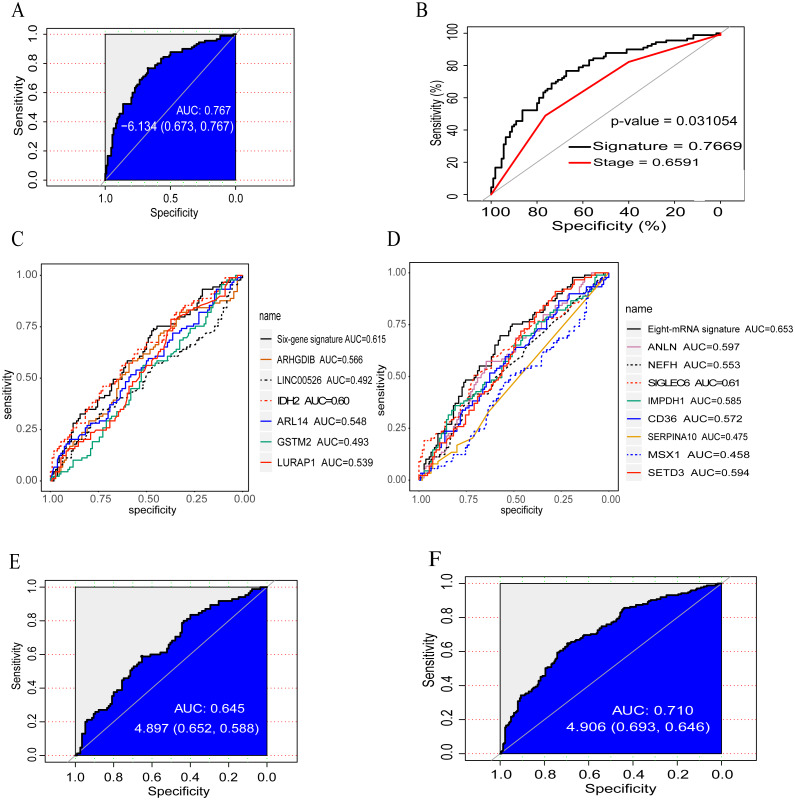
Evaluation of the predictive power of the PCG-lncRNA-microRNA signature and pathologic stage in the training set, test set and entire set. (A) ROC analysis of the signature for prediction of overall survival in the training set. (B) Comparison of the survival prediction ability of the PCG-lncRNA-microRNA signature with pathologic stage in the training set. (C) ROC curves of the six-gene signature. (D) ROC curves of the eight-mRNA signature. (E) ROC analysis of the signature for prediction of overall survival in the test set. (F) ROC analysis of the signature for estimation of overall survival in the entire set.

The 3 PCGs, 3 lncRNAs and 3 microRNAs mentioned above in the training set could have 511 combinations and the corresponding risk score could be calculated according to every risk score model (Table S2). All of the risk scores of every patient were computed in the “Risk score (RS)” formula described in the methods. Simultaneously, we conducted ROC analysis for selecting a better prognostic signature. Finally, the PCG-lncRNA-microRNA model composed of *ANXA1* (PCG), *TPST1* (PCG), *PSMB10* (PCG), *DLEU1* (lncRNA) and *miR-497-5p* (miRNA) with a higher AUC and the significant regression coefficients (*p*-value < 0.05) was retained as the ultimate model (Table 2). The risk score of each patient was obtained with the criteria outlined below: Risk score = (0. 3024 × expression value of *ANXA1*) + (0. 1494 × expression value of *TPST1*) + (−0. 4377 × expression value of *PSMB10*) + (0. 4875 × expression value of *DLEU1*) + (0. 4262 × expression value of *miR-497-5p*). The AUC value of the PCG-lncRNA-microRNA signature mentioned above was 0.7669 (Fig. 2A), indicating that the model consisting of the five biomarkers could have a good performance for survival prediction. We also compared the survival predictive ability of the PCG-lncRNA-microRNA signature with pathologic stage using ROC analysis in the training dataset and concluded that the predictive power of the signature constructed was better than stage (AUC_signature_ = 0.7669, AUC_stage_ = 0.6591; *p*-value < 0.05) (Fig. 2B). In this study, we also compared the proposed model with other known prognostic signatures, including a six-gene signature (Wang et al., 2019) and an eight-mRNA signature (Zhu et al., 2019). As shown in the Figs. 2C–2D, the AUC value of the model proposed in this study was larger than the studies mentioned above. The result indicated that the prognostic signature model was candidate for predicting patients’ overall survival status. The results further indicated that the model constructed in our study was a novel predictive prognostic signature with high sensitivity and specificity in its clinical significance. Furthermore, from the “Risk score” formula, it suggested that *ANXA1*, *TPST1*, *DLEU1* and *miR-497-5p* were possible risk factors and *PSMB10* was a possible protective factor for survival (Table 2).

**Table 2:** Identifications of PCGs, lncRNA and microRNA in the prognostic expression signature.

**Gene symbol**	**Gene name**	**Gene type**	**Location**	**Coefficient**^a^	**HR**	**lower95**	**upper95**	***p*-value**^b^
ANXA1	Annexin A1	Protein-coding	9q21.13	0.3024	1.3531	1.1664	1.5696	<0.0001
TPST1	Tyrosylprotein sulfotransferase 1	Protein-coding	7q11.21	0.1494	2.183	1.416	3.365	<0.0001
PSMB10	Proteasome subunit beta 10	Protein-coding	16q22.1	−0.4377	0.6455	0.5169	0.8061	0.000113
DLEU1	Deleted in lymphocytic leukemia 1	lncRNA	13q14.2-q14.3	0.4875	1.6283	1.2103	2.1907	0.001279
miR-497-5p	MIR497	microRNA	17p13.1	0.4262	1.5314	1.1825	1.9833	0.001237

**Notes.**

HR, hazard ratio.

a Coefficents derived from multivariate Cox regression analysis.

b *p*-values obtained from multivariate Cox regression analysis.

**Figure 3:**
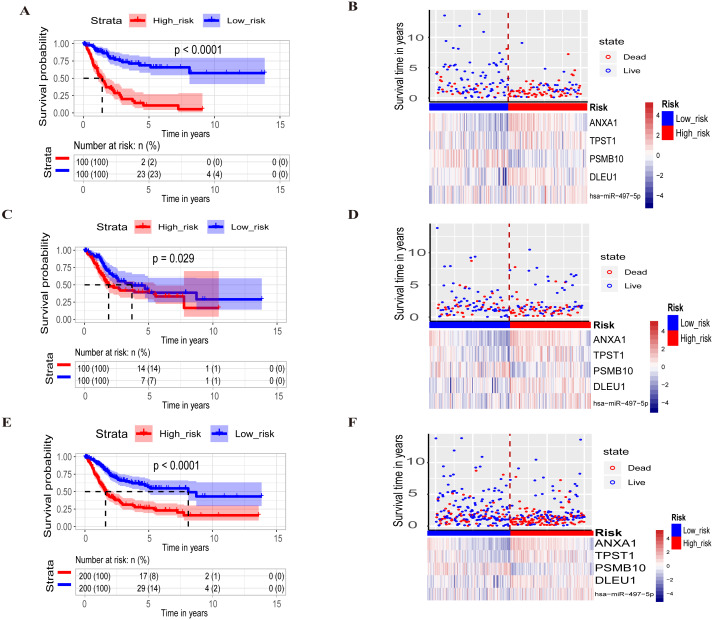
Screening and constructing of the prognostic PCG-lncRNA-microRNA signature model. (A) Kaplan–Meier analysis for overall survival in BLCA patients stratified according to the five-RNA signature into high-risk and low-risk groups in the training set. Similar results were presented in the test set (C) and in the entire set (E). (B) The distribution of risk scores, OS, vital status, and corresponding RNAs expression profiles of the 200 patients in the high-risk and low-risk groups in the training set. Similar results were presented in the test set (D) and in the entire set (F). The “+” symbol in the panel indicated censored data. Risk scores are presented and arranged in ascending order from left to right in the x-axis. The vital status is shown with red and green spots, respectively. Heatmaps of RNA expression profiles of the selected five biomarkers in the high- and low- groups according to risk scores.

The 200 BLCA patients in the training set were assigned to the high-risk group (*n* = 100) and low-risk group (*n* = 100) according to the median risk score. Kaplan–Meier analysis indicated that patients in the high-risk group had a significantly poor outcome than those in the low-risk group (log-rank test, *p*-value < 0.0001; Fig. 3A). The distribution of risk scores, OS, vital status, and corresponding RNAs expression profiles of the 200 patients in the high-risk and low-risk groups are shown in Fig. 3B.

### Validation of the prognostic signature model in the test set

Using the same method as in the training set, the patients in the test set (*n* = 200) and entire set (*n* = 400) were also classified into high-risk and low-risk groups to validate the survival prediction of the PCG-lncRNA-microRNA signature. In the test set, the 200 patients were divided into the high-risk group (*n* = 100) and low-risk group (*n* = 100). Kaplan–Meier curve analysis showed that patients in the high-risk group had a shorter OS than those in the low-risk group (log-rank test, *p*-value < 0.05; Fig. 3C). The AUC value of the signature was 0.645 (Fig. 2E). In the entire set, similar results were found (log-rank test, *p*-value < 0.0001; Fig. 3E). The AUC of the signature was 0.710 (Fig. 2F). However, the entire set was just used for exploration purposes instead of evaluating the value for the model. The distribution of risk scores, OS, vital status, and corresponding RNAs expression profiles of patients in the test set and entire set in the high-risk and low-risk groups are shown in Fig. 3D and Fig. 3F. We also noticed that *ANXA1*, *TPST1*, *DLEU1* and *miR-497-5p* showed a tendency towards high expression in patients in the high-risk group, and *PSMB10* presented with low expression in the high-risk group. These results are in accordance with the results in the training set (please see the section above) (Figs. 3D–3F).

### The survival predictive ability of the three-dimension transcriptome signature is independent of other clinical features

**Figure 4:**
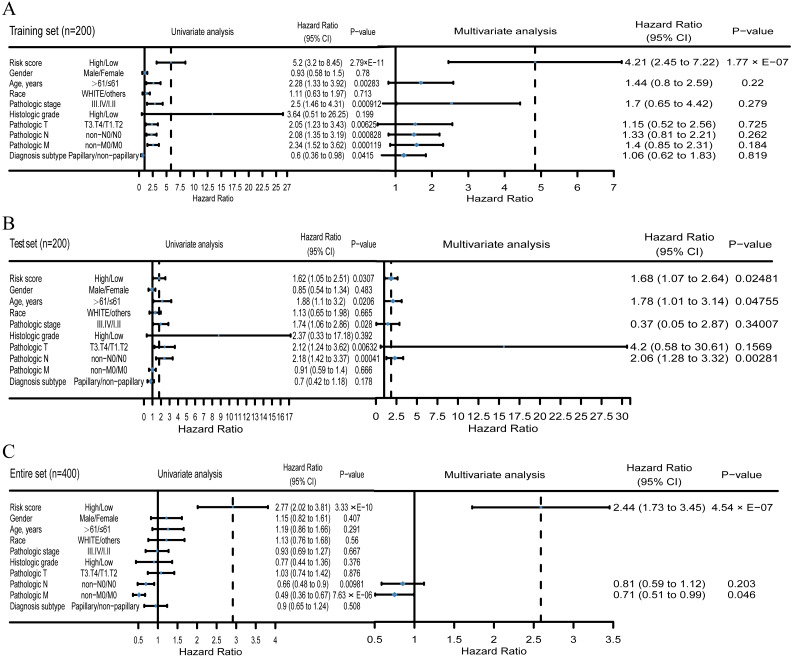
Univariate and multivariate Cox regression analysis of the PCG-lncRNA-microRNA signature and overall survival of BLCA patients in the training (A), test (B) and entire set (C).

To evaluate whether the PCG-lncRNA-microRNA signature maintained its prognostic power in the context of other clinical features, the results of multivariate Cox regression analysis showed that the power of the three-dimension transcriptome signature was maintained in survival predication and was significantly independent of other clinical features in the training set (hazard ratio [HR] = 4.209, 95% confidence interval [CI] [2.4541–7.218], *p*-value < 0.0001), the test set (HR = 1.679, 95% CI [1.0679–2.641], *p*-value < 0.05), and the entire set (HR = 2.4419, 95% CI [1.7262–3.454], *p*-value < 0.0001; Fig. 4).

### NMF and SNF algorithm identifies three subtypes in BLCA

NMF was applied to the training set and cophenetic correlation coefficients were calculated to choose the appropriate number of clusters. Ultimately, a factorization rank of 3 was determined for the clusters by the method mentioned by Gaujoux & Seoighe (2010) who suggested choosing the smallest value of r for which this coefficient starts decreasing (Fig. S2A). The consensus matrix heatmap showed the preferable sharp boundaries, which indicated robust and stable clustering for the samples (Fig. S2B).

Then, 172 PCGs, 15 lncRNAs and 42 miRNAs metagenes identified by NMF were described as features, together with the 200 samples in the training set to build a similarity network to cluster cancer subtypes based on three-dimension transcriptome data. We classified all of the 200 BLCA patients into three clusters: cluster_1 (63 patients, 31.5%), clusters_2 (85 patients, 42.5%) and clusters_3 (52 patients, 26.0%). Statistical significance analysis of clustering showed that cluster_1/cluster_2, cluster_1/cluster_3 and cluster_2/cluster_3 had significant differences (*p*-value = 0) (Fig. S2C). The same classification method was performed in the test set and the entire set. Patients in these two datasets can also be divided into three clusters that presented with similar proportions (Fig. S2D). Furthermore, we found that patients in the high-risk group with high grade and stage I; and stage II; were enriched in subtype 2 and subtype 3 and few were in subtype 1, while patients in the low-risk group were enriched in subtype 1 and subtype 2 and few were in subtype 3. This suggested that subtype 2 and subtype 3 have potentially higher carcinogenic biological processes (Figs. S2E–S2G).

**Figure 5:**
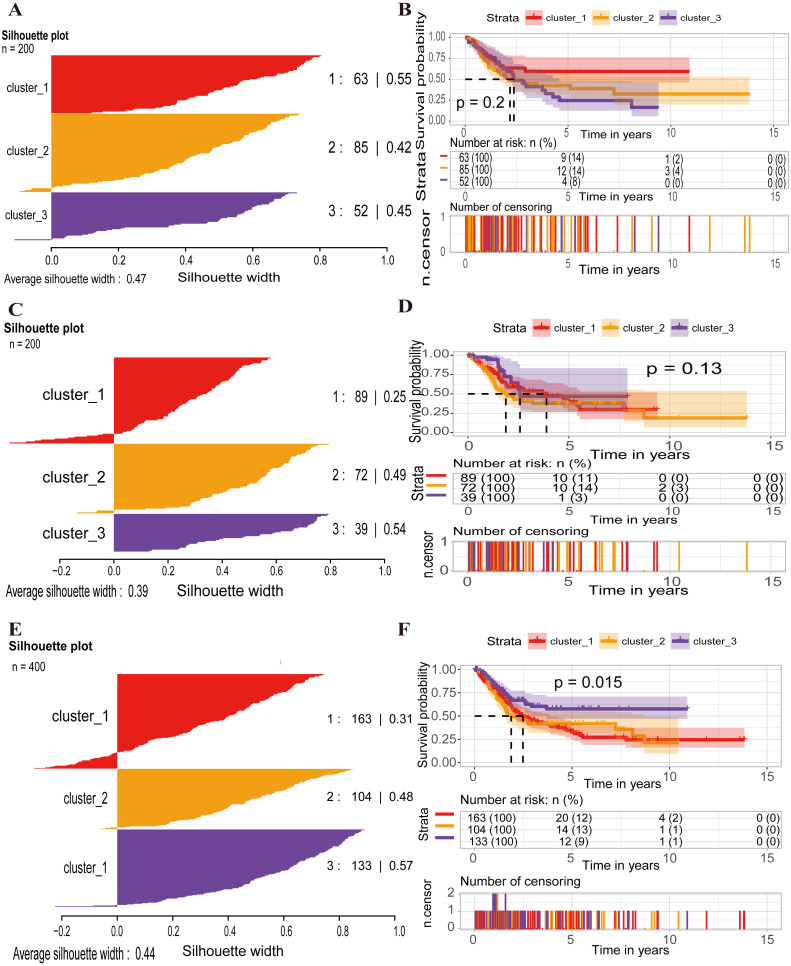
Classification of BLCA into three molecular subtypes. Silhouette information for *k* = 3 classes and Kaplan–Meier survival curve comparing the survival of cluster_1 (red), cluster_2 (orange), and cluster_3 (purple) in the training set (A), test set (C) and entire set (E). Survival differences were calculated using the log-rank test, respectively (B, D, F). *P*-values of less than 0.05 were considered statistically significant.

The average silhouette width between these clusters was 0.47 (range, from 0.42 to 0.55), which indicated the robustness of the classification in the training set (Fig. 5A). However, the overall survival probability between the three clusters had no significant differences (*p*-value = 0.2) (Fig. 5B). Meanwhile, the high-risk group had a poor prognosis compared with the low-risk group in every cluster (cluster_1: *p*-value < 0.0001, cluster_2: *p*-value < 0.0001, cluster_3: *p*-value = 0.00049) (Fig. S3). In the test set, the average silhouette width between the three clusters was 0.39 (range, from 0.25 to 0.54), the OS probability across the three clusters had no significant differences (*p*-value = 0.13) (Figs. 5C–5D), and the survival probability between the high-risk group and low-risk group had no significance except for the cluster_2 (*p*-value = 0.0085) (Fig. S3). In the entire set, the average silhouette width across the three clusters was 0.44 (range, from 0.31 to 0.57), the entire probability across the three clusters had significant differences (*p*-value = 0.015) (Figs. 5E–5F), while the high-risk group had a poor prognosis compared with the low-risk group in every cluster (cluster_1: *p*-value < 0.0001, cluster_2: *p*-value = 0.00059, cluster_3: *p*-value < 0.0001) (Fig. S3). The heatmap was presented in Fig. S4. The basal squamous, neuronal, luminal papillary and luminal infiltrated subtypes shared in the same cluster 3, which showed higher TP53 mutation and more dead events. And the majority of basal squamous and neuronal subtypes were enriched in the cluster 3, indicating high risk group, while the luminal papillary subtype was enriched in the cluster 2. Furthermore, the lncRNA and microRNA clusters also presented preferable general boundaries.

We also investigated the basic expression of these five biomarkers in the tumor and normal tissues (Fig. S5A). Based on the biomarkers of the constructed signature, we also investigated the potential biological significance behind these molecules. We calculated the expression correlation of the 5 genes, and the correlation coefficients of most of the genes were low in the subtypes or tumour samples. The genes with positive regression coefficients had positive correlations with each other. The relationship of *ANXA1* and *TPST1* showed a positive correlation (*r* = 0.21, *p*-value < 0.05) and the association of *ANXA1* and *DLEU1* also showed a positive correlation (*r* = 0.21, *p*-value < 0.05) (Fig. S5B). All of these indicated that these genes carried less overlapped information and showed low redundancy.

### Functional characterization of GO and KEGG analyses and gene set variation analysis (GSVA)

**Figure 6:**
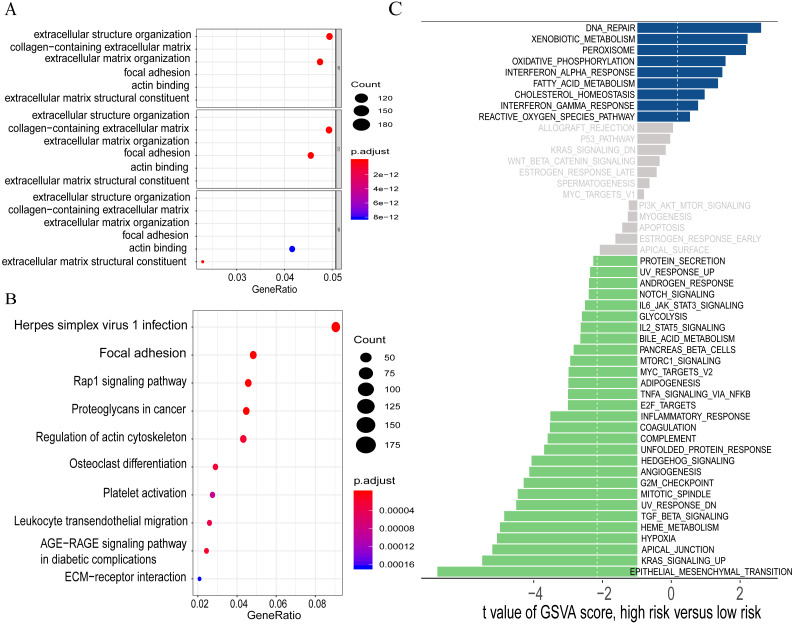
Visualization for the results of GO (A), KEGG (B) and GSVA analyses (C).

To explore the co-expression relationships of the selected PCGs, lncRNAs and miRNAs with the PCGs, Pearson correlation coefficients were computed according to the standard. The expression of 4469/8969 protein-coding genes in the training set were highly correlated with that of at least one of the biomarkers. GO analysis, including biological process, cellular component and molecular function, was conducted. Focal adhesion and MAPK signaling pathway were also identified in the KEGG analysis. Then, GSVA for these co-expressed PCGs was performed based on the Hallmark gene sets from the Molecular Signatures Database (MSigDB). Several functionally related terms were identified and suggested that the selected five signatures might be involved in tumourigenesis by interacting with related PCGs that referred to important biological processes such as “epithelial to mesenchymal transition” (EMT), “KRAS signaling up” and so on (Fig. 6; Table S3).

Furthermore, the protein of the prognostic markers shared in the same subcellular localization, including plasma membrane, extracellular, nucleus and endosome and so on (Table S3). Data were from a subcellular localization database COMPARTMENTS (https://compartments.jensenlab.org/Search).

## Discussion

Heterogeneity makes cancers not just a single disease but a diverse group of diseases and this presents a large significant difficulty and challenge to the treatment of cancer patients. With the ability to perform genome-wide molecular profiling of cancers and the increasing prevalence of high-throughput sequencing technology in biological studies, especially in the development of gene expression profiling technologies, researchers can confront the genetic challenge to better understand the heterogeneity of a variety of cancers. Bladder urothelial carcinoma accounts for 90% of bladder cancers, and tumour node metastasis (TNM) classification and pathological grade are incapable of adequately and precisely forecasting their patients’ clinical outcomes. In the development of molecular technologies, it is urgent to identify and validate a novel biomarker accounting for molecular mechanisms of bladder urothelial carcinoma and predicting individual survival of patients. Meanwhile, heterogeneity existing for each tumour type makes it hard to select the right treatment strategies for bladder cancer patients with different molecular subtypes. The evidence has shown that one single RNA element is less sensitive and specific for clinical outcomes than a combination of multi-dimensional levels (Xu et al., 2017). We believed that the three-dimension transcriptome signature represented the combination of three types of RNAs, including protein-coding RNA, lncRNA and microRNA. Hence, we constructed a risk score formula based on a signature composed of PCGs, lncRNAs and miRNAs, which are involved in oncogenic and tumour suppressive pathways and that may show a predictive power in BLCA patients.

In this comprehensive analysis, we adopted various algorithms to identify three PCGs, one lncRNA and one miRNA whose expression levels were associated with the overall survival of bladder urothelial carcinoma patients. Then, we further unveiled that the signature created by combining these biomarkers was related to BLCA patient clinical outcomes. The multi-dimensional RNA signature could effectively divide patients in the training set into high- and low- groups with significantly different overall survival, and this finding could be successfully validated in the entire set and show a marginally significant difference in the test set. We found that the AUC value was low in the test set, and the KM-curve in the test set is only weakly significant ( *p* = 0.03), and the curves separate marginally in the first years of follow-up but eventually meet again after 5 years. While, it is the metric that 5-year survival after cystectomy is clinically most valuable because few additional events occur after 5 years, so this metric is the key factor on whether the patient was cured by the surgery, or died by the cancer. These suggested that the performance of our model was to some degree data set dependent. However, only this weak prognostication could be achieved despite optimal combinatorial analysis of many thousands of variables. This is saying something important about the (low) amount of prognostic information that is found in data sets of muscle-invasive bladder cancer. Moreover, with clinical covariates in the multivariate Cox regression model, the signature identified in this study was assessed to be to some extent an independent factor for predicting OS in BLCA patients. Ethnicity (Yu, Qian & Yang, 2018) and gender (Marcus et al., 2008) have been suggested to influence the prevalence of the disease and the survival of the bladder cancer patients. Yu et al. considered that non-Hispanic white patients have the highest incidence rate and better survival rates. However, in this study, we concluded that there was no significant difference of the overall survival between the white patients and the other ethnicity in our univariate analysis. Differences in results may account for the small sample size (*n* = 200) and lack of statistical power, and also the gender. Taken together, these results suggested that the constructed PCG-lncRNA-miRNA signature might be beneficial for clinical identification of and selection of therapeutic strategies for patients who are experiencing pain and need more treatment to prolong their lives. But more datasets were needed to verify the model. In addition, compared to the study of Robertson et al., we integrated three types of multi-omics to predict overall survival, and the screening process included different sophisticated algorithms.

Likewise, compared with the traditional classification of cancers, categorical methods based on transcriptomes can be utilized to classify cancer samples into subtypes with different molecular characteristics and clinical significance. We applied NMF to perform gene expression profiling for the classification of BLCA. We identified three molecular and clinical clusters in the training set and the high-risk group had a poor prognosis compared with the low-risk group in every cluster. These results suggested that BLCA is a highly heterogeneous disease and demonstrated that transcriptome expression profiling applied for categorization of cancers has molecular and clinical significance.

The initiation and progression of BLCA requires the activation of potential key signalling pathways and dysregulation of cellular biological processes. Gene set variation analysis of co-expression protein-coding genes of the five biomarkers of the signature showed that these genes were enriched in the processes and pathways of tumourigenesis in BLCA. Accumulating evidence demonstrated that these discovered biomarkers play crucial roles in a variety of human cancers. Annexin A1 (*ANXA1*) is a protein-coding gene and encodes a membrane-localized protein that binds to phospholipids. *ANXA1* has been reported to have an anti-proliferative effect mediated by the intracellular form of the protein, and it has been found that both mRNA and protein levels are down-regulated in head and neck cancer tissues*. ANXA1* is overexpressed in familial breast cancer patients with *BRCA1/2* mutations and is associated with poor prognostic features such as triple negative and poorly differentiated tumors and may be biomarker candidates for breast cancer survival prediction in high risk populations such as HER2+ cases (Sobral-Leite et al., 2015). Researchers have concluded that *ANXA1* acts as a tumour suppressor in head and neck squamous cell carcinoma (HNSCC) and could be an important prognostic biomarker (Raulf et al., 2018).

Studies depicting the function of tyrosylprotein sulfotransferase 1 (*TPST1*) in cancer are rare. In tumour tissues, *TPST1* appears to be significantly lower expression than in control lung tissues. The *TPST 1* expression was significantly associated with lymph node metastasis and the tumour node metastasis (TNM) stage in patients with lung cancer and may be a negative prognostic biomarker of lung cancer (Jiang et al., 2015). Upregulation of *TPST-1* might be an underlying mechanism contributing to NPC metastasis (Xu et al., 2013).

Proteasome subunit beta 10 (*PSMB10*), known as the immunoproteasome (IP) gene, is a multi-catalytic proteinase complex with a highly ordered ring-shaped 20S core structure. In breast cancer, high expression of the IP gene is associated with a longer survival. In contrast, IP upregulation is a cell-intrinsic feature that is not associated with longer survival in acute myeloid leukaemia (AML). Especially, in M5 AML, expression of the IP gene was found to be mostly co-regulated with genes involved in mitochondrial activity and stress responses, cell metabolism and proliferation (Rouette et al., 2016).

Deleted in lymphocytic leukaemia 1 (*DLEU1*), as a long-noncoding RNA, has potential mechanisms underlying tumourigenesis. In colorectal cancer tissues, by activating *KPNA3* via recruiting *SMARCA1*, an essential subunit of the NURF chromatin remodelling complex, increased expression of *DLEU1* was observed, and higher expression of *DLEU1* in patients indicated lower survival rate and a poorer prognosis (Liu et al., 2018a). In oral squamous cell carcinoma (OSCC) cells, *DLEU1* has oncogenic functionality and participates in migration, invasion, and xenograft formation. Elevated *DLEU1* expression contributes to OSCC progress and high *DLEU1* expression has been associated with shorter overall survival of primary head and neck squamous cell carcinoma patients (Nishiyama et al., 2018). Higher lncRNA-*DLEU1* expression is found in epithelial ovarian carcinoma (EOC) tissues than in normal tissues. In the ovarian cancer cell lines A2780 and OVCAR3, plasmid transfection of *DLEU1* to upregulate its expression increased cell proliferation, migration, and invasion while inhibiting apoptosis (Wang et al., 2017).

Research on *miR-497-5p* in human cancers reported that the expression of *miR-497-5p* was lower in cancer tissues than in normal tissues in HPV-infected patients with cervical cancer in the Uyghur population in China (Gao et al., 2016). Overexpression of *miR-497-5p* inhibited A375 cell proliferation, migration and invasion, arrested the cell cycle, induced cell apoptosis, and it decreased *hTERT* expression at both the mRNA and protein levels. *MiR-497-5p* acts as tumour suppressor by targeting *hTERT* in melanoma A375 cells (Chai et al., 2018).

In this study, we found that *TPST1* and *ANXA1* were considered as risk factors while *PSMB10* was a protective factor. Deeper research into these two genes has potential value. In addition, molecular subtyping of BLCA in our study may be helpful for selecting specific treatment strategies for patients in different subgroups.

Nevertheless, the current study also has some limitations presented as follows. First, although mathematical algorithms are a powerful method of identifying the potential biological mechanisms behind high density data, further in vivo/in vitro experiments to verify the identified biomarkers are still needed to provide more convincing explanations of the biological evidence. Second, the method of the choice of 50/50 training and test sets may be outdated, and the training set can be significantly bigger to capture more information. Cross-validation may be alternative. However, in this study, we adopted the “sample” algorithm, this algorithm was generating random samples. Therefore, it is urgent to expand the sample size for verification. Furthermore, as a retrospective study, the cohort of patients was heterogeneous, and the number of tissues derived from one database (TCGA) was limited, and as a result, the robustness of the results in prognostic assessment must be validated in prospective patient cohorts in clinical trials or external validation datasets, ideally with large prospective patient cohorts. We have tried our best to search for more datasets in many kinds of databases, but it is very hard to find out the clinical samples simultaneously including survival information and all expression data of the protein-coding genes (PCGs), lncRNA and microRNA, which were included in our proposed model. Third, the number of lncRNAs and miRNAs screened in the gene expression profiling in this study was rare, and as a result, lncRNAs and miRNAs were seldom included in our established model. The signature might not represent all of the candidate biomarkers that are potentially associated with survival of bladder urothelial carcinoma. Fourth, the data shows that despite sophisticated gene selection and modeling, it was not possible to get a good separation of survival in the test set. The appropriate weighting of the genes is to some degree method and data set dependent. When the model was tested in NMF-clusters, which are a representation of RNA-based molecular subtypes, they failed to provide any survival information in the individual subsets. More genes, or a different selection strategy e.g., knowledge driven, or using coherent signatures, or subtype-dependent signatures may be better approaches for survival prediction. Moreover, molecular categorization is a whole field of tumour research that can not be necglected, and to obtain the precise classification of the tumors, multi-omics data and various algorithms were need to draw preferable subgroups. Fifth, the analysis excluded the samples with missing overall survival data. Missing data in general, including the overall survival, may affect the survival models and predictions, however the impact of missing death data on survival analyses and estimates of overall survival is small when mortality capture sensitivity is high (e.g., approximately 90% or more) (Carrigan et al., 2019).

## Conclusions

All in all, in a comprehensive analysis, we have established a PCG-lncRNA-miRNA signature that has to some degree the independent power of predicting overall survival of bladder urothelial carcinoma patients. However, despite sophisticated gene selection and modeling, the verification in the test set is only weakly significant, and the appropriate weighting of the genes is to some degree method and data set dependent. The findings of this proposed model could have value in the introduction of personalized therapies. Furthermore, we also identified three molecular subtypes in patients with BLCA, and the constructed signature could stratify the risk of OS among patients in every subtype. However, large-scale clinical trials and replication experiments are required to assess the possible molecular signature to predict survival.

##  Supplemental Information

10.7717/peerj.9422/supp-1 Supplemental Information 1 Random forests for survival, regression, and classification analysis reveals the variable importance of corresponding RNAs

10.7717/peerj.9422/supp-2 Supplemental Information 2 The molecular subtypes identification based on NMF and SNF in the training set (A) Unsupervised classification and selection of the appropriate cophenetic correlation coefficient using NMF. A factorization rank of k = 3 was determined as the optimal number of clusters (k). (B) NMF clustering was performed 200 times with optimal k to obtain the NMF consensus matrix. (C) Statistical significance of clustering (SigClust) was performed to validate the significant difference of clustering results in the mRNA expression. (D) Patient distribution into the training set (*n* = 200), test set ( *n* = 200) and entire set (*n* = 400). (E) The correlation of the risk groups, subtypes and clinical status. Distribution of the clinical features, including neoplasm_histologic_grade (F) and pathologic_stage (G) in the identified three clusters.

10.7717/peerj.9422/supp-3 Supplemental Information 3 Kaplan–Meier survival curve comparing the survival in the high- and low- risk groups in every cluster in the training set, test set and entire set, respectively

10.7717/peerj.9422/supp-4 Supplemental Information 4 The heatmap corresponding to the dendrogram for the meta-genes classification in the entire set The heatmap was generated using the heatmap function with SNF algorithm classification, mRNA cluster, lncRNAcluster, microRNA cluster, RPPA cluster, hypermethylation cluster,hypomethylation cluster, mutation process (MSig) cluster, SMG-SCNA cluster, histological subtype, gender, survival status, TNM stages, clinicopathologicalstages, histological grade, and TP53 mutation, KRAS mutation, BRAF mutation, EGFR mutation as the annotations.

10.7717/peerj.9422/supp-5 Supplemental Information 5 (A) The basic expression of the five prognostic biomarkers in TCGA. (B) The correlative features of the five prognostic RNAs. The expression correlation analyses of the five feature genes in the whole training set and the subtypes in the training set and normal group. The diagonal is the expression distribution mount of each of the genes; the lower left corner is the gene expression level of the scatter diagram between the two corresponding genes; the upper right corner part is the correlation coefficient of every two genes (ranging from –1 to +1). The significance of the correlation was labelled with “*” (*p*-value < 0.05) and “**” (*p*-value < 0.01).

10.7717/peerj.9422/supp-6 Supplemental Information 6 Univariate Cox regression analysis of PCGs, lncRNAs and microRNAs in the training set (*n* = 200)

10.7717/peerj.9422/supp-7 Supplemental Information 7 The signature composed of PCGs, lncRNAs and microRNAs in the training (*n* = 200), test set (*n* = 200) and entire set (*n* = 400)

10.7717/peerj.9422/supp-8 Supplemental Information 8 GO, KEGG, gene set variation analysis (GSVA) and subcellular localization information

## Additional Information and Declarations

### Data Availability

The following information was supplied regarding data availability: The raw measurements are available in the UCSC Xena portal (https://xenabrowser.net/datapages/) using the search term: “TCGA Bladder Cancer (BLCA)”. All of the R codes in this study are availale at Github: https://github.com/liudanqi05/BLCA_signature).

## References

[ref-1] Bao Z, Zhang W, Dong D (2017). A potential prognostic lncRNA signature for predicting survival in patients with bladder urothelial carcinoma. Oncotarget.

[ref-2] Berger AC, Korkut A, Kanchi RS, Hegde AM, Lenoir W, Liu W, Liu Y, Fan H, Shen H, Ravikumar V, Rao A, Schultz A, Li X, Sumazin P, Williams C, Mestdagh P, Gunaratne PH, Yau C, Bowlby R, Robertson AG, Tiezzi DG, Wang C, Cherniack AD, Godwin AK, Kuderer NM, Rader JS, Zuna RE, Sood AK, Lazar AJ, Ojesina AI, Adebamowo C, Adebamowo SN, Baggerly KA, Chen TW, Chiu HS, Lefever S, Liu L, MacKenzie K, Orsulic S, Roszik J, Shelley CS, Song Q, Vellano CP, Wentzensen N, Weinstein JN, Mills GB, Levine DA, Akbani R (2018). A comprehensive pan-cancer molecular study of gynecologic and breast cancers. Cancer Cell.

[ref-3] Bircan HA, Gurbuz N, Pataer A, Caner A, Kahraman N, Bayraktar E, Bayraktar R, Erdogan MA, Kabil N, Ozpolat B (2018). Elongation factor-2 kinase (eEF-2K) expression is associated with poor patient survival and promotes proliferation, invasion and tumor growth of lung cancer. Lung Cancer.

[ref-4] Brunet JP, Tamayo P, Golub TR, Mesirov J (2004). Metagenes and molecular pattern discovery using matrix factorization. Proceedings of the National Academy of Sciences of the United States of America.

[ref-5] Buffa FM, Camps C, Winchester L, Snell CE, Gee HE, Sheldon H, Taylor M, Harris AL, Ragoussis J (2011). microRNA-associated progression pathways and potential therapeutic targets identified by integrated mRNA and microRNA expression profiling in breast cancer. Cancer Research.

[ref-6] Cai H, Lin L, Cai H, Tang M, Wang Z (2013). Prognostic evaluation of microRNA-210 expression in pediatric osteosarcoma. Medical Oncology.

[ref-7] Camp RL, Dolled-Filhart M, Rimm DL (2004). X-tile: a new bio-informatics tool for biomarker assessment and outcome-based cut-point optimization. Clinical Cancer Research.

[ref-8] Carrigan G, Whipple S, Taylor MD, Torres AZ, Gossai A, Arnieri B, Tucker M, Hofmeister PP, Lambert P, Griffith SD, Capra WB (2019). An evaluation of the impact of missing deaths on overall survival analyses of advanced non- small cell lung cancer patients conducted in an electronic health records database. Pharmacoepidemiology Drug Safety.

[ref-9] Chai L, Kang XJ, Sun ZZ, Zeng MF, Yu SR, Ding Y, Liang JQ, Li TT, Zhao J (2018). MiR-497-5p, miR-195-5p and miR-455-3p function as tumor suppressors by targeting hTERT in melanoma A375 cells. Cancer Management and Research.

[ref-10] Eilertsen M, Andersen S, Al-Saad S, Richardsen E, Stenvold H, Hald SM, Al-Shibli K, Donnem T, Busund LT, Bremnes RM (2014). Positive prognostic impact of miR-210 in non-small cell lung cancer. Lung Cancer.

[ref-11] Fan CN, Ma L, Liu N (2018). Systematic analysis of lncRNA-miRNA-mRNA competing endogenous RNA network identifies four-lncRNA signature as a prognostic biomarker for breast cancer. Journal of Translational Medicine.

[ref-12] Gao D, Zhang Y, Zhu M, Liu S, Wang X (2016). miRNA expression profiles of HPV-infected patients with cervical cancer in the uyghur population in China. PLOS ONE.

[ref-13] Gaujoux R, Seoighe C (2010). A flexible R package for nonnegative matrix factorization. BMC Bioinformatics.

[ref-14] Ge Y, Zhang C, Xiao S, Liang L, Liao S, Xiang Y, Cao K, Chen H, Zhou Y (2018). Identification of differentially expressed genes in cervical cancer by bioinformatics analysis. Oncology Letters.

[ref-15] Gee HE, Camps C, Buffa FM, Patiar S, Winter SC, Betts G, Homer J, Corbridge R, Cox G, West CM, Ragoussis J, Harris AL (2010). hsa-mir-210 is a marker of tumor hypoxia and a prognostic factor in head and neck cancer. Cancer.

[ref-16] Grambsch PM, Therneau TM (2000).

[ref-17] Greither T, Grochola LF, Udelnow A, Lautenschlager C, Wurl P, Taubert H (2010). Elevated expression of microRNAs 155, 203, 210 and 222 in pancreatic tumors is associated with poorer survival. International Journal of Cancer.

[ref-18] Greither T, Wurl P, Grochola L, Bond G, Bache M, Kappler M, Lautenschlager C, Holzhausen HJ, Wach S, Eckert AW, Taubert H (2012). Expression of microRNA 210 associates with poor survival and age of tumor onset of soft-tissue sarcoma patients. International Journal of Cancer.

[ref-19] Han Y, Zheng Q, Tian Y, Ji Z, Ye H (2019). Identification of a nine-gene panel as a prognostic indicator for recurrence with muscle-invasive bladder cancer. Journal of Surgical Oncology.

[ref-20] Hanzelmann S, Castelo R, Guinney J (2013). GSVA: gene set variation analysis for microarray and RNA-seq data. BMC Bioinformatics.

[ref-21] Hess J, Unger K, Maihoefer C, Schuttrumpf L, Wintergerst L, Heider T, Weber P, Marschner S, Braselmann H, Samaga D, Kuger S, Pflugradt U, Baumeister P, Walch A, Woischke C, Kirchner T, Werner M, Werner K, Baumann M, Budach V, Combs SE, Debus J, Grosu AL, Krause M, Linge A, Rodel C, Stuschke M, Zips D, Zitzelsberger HF, Ganswindt U, Henke M, Belka C (2019). A five-microRNA signature predicts survival and disease control of patients with head and neck cancer negative for hpv-infection. Clinical Cancer Research.

[ref-22] Hyung J, Cho A, Yu SK (2009). Robust likelihood-based survival modeling with microarray data. Journal of Statistical Software.

[ref-23] Inamoto T, Uehara H, Akao Y, Ibuki N, Komura K, Takahara K, Takai T, Uchimoto T, Saito K, Tanda N, Yoshikawa Y, Minami K, Hirano H, Nomi H, Kato R, Hayashi T, Azuma H (2018). A Panel of microRNA signature as a tool for predicting survival of patients with urothelial carcinoma of the bladder. Disease Markers.

[ref-24] Ishwaran H, Gerds TA, Kogalur UB, Moore RD, Gange SJ, Lau BM (2014). Random survival forests for competing risks. Biostatistics.

[ref-25] Ishwaran H, Kogalur UB (2010). Consistency of random survival forests. Statistics & Probability Letters.

[ref-26] Jiang B, Hailong S, Yuan J, Zhao H, Xia W, Zha Z, Bin W, Liu Z (2018). Identification of oncogenic long noncoding RNA SNHG12 and DUXAP8 in human bladder cancer through a comprehensive profiling analysis. Biomedicine and Pharmacotherapy.

[ref-27] Jiang Z, Zhu J, Ma Y, Hong C, Xiao S, Jin L (2015). Tyrosylprotein sulfotransferase 1 expression is negatively correlated with cMet and lymph node metastasis in human lung cancer. Molecular Medicine Reports.

[ref-28] Kamat AM, Hahn NM, Efstathiou JA, Lerner SP, Malmstrom PU, Choi W, Guo CC, Lotan Y, Kassouf W (2016). Bladder cancer. Lancet.

[ref-29] Kendall WL, Pollock KH, Brownie C (1995). A likelihood-based approach to capture-recapture estimation of demographic parameters under the robust design. Biometrics.

[ref-30] Liu T, Han Z, Li H, Zhu Y, Sun Z, Zhu A (2018a). LncRNA DLEU1 contributes to colorectal cancer progression via activation of KPNA3. Molecular Cancer.

[ref-31] Liu J,  Lichtenberg T, Hoadley KA, Poisson LM, Lazar AJ, Cherniack AD, Kovatich AJ, Benz CC, Levine DA, Lee AV, Omberg L,  Wolf DM, Shriver CD, Thorsson V, Hu H (2018b). An integrated TCGA pan-cancer clinical data resource to drive high-quality survival outcome analytics. Cell.

[ref-32] Liu G, Zheng J, Zhuang L, Lv Y, Zhu G, Pi L, Wang J, Chen C, Li Z, Liu J, Chen L, Cai G, Zhang X (2018c). A prognostic 5-lncRNA expression signature for head and neck squamous cell carcinoma. Scientific Reports.

[ref-33] Liu R, Zhang W, Liu ZQ, Zhou HH (2017). Associating transcriptional modules with colon cancer survival through weighted gene co-expression network analysis. BMC Genomics.

[ref-34] Marcus H, Ralf W, Markus F, Arnulf S (2008). Gender-specific differences in bladder cancer: a retrospective analysis. Comparative Study.

[ref-35] McCormick RI, Blick C, Ragoussis J, Schoedel J, Mole DR, Young AC, Selby PJ, Banks RE, Harris AL (2013). miR-210 is a target of hypoxia-inducible factors 1 and 2 in renal cancer, regulates ISCU and correlates with good prognosis. British Journal of Cancer.

[ref-36] Nishiyama K, Maruyama R, Niinuma T, Kai M, Kitajima H, Toyota M, Hatanaka Y, Igarashi T, Kobayashi JI, Ogi K, Dehari H, Miyazaki A, Yorozu A, Yamamoto E, Idogawa M, Sasaki Y, Sugai T, Tokino T, Hiratsuka H, Suzuki H (2018). Screening for long noncoding RNAs associated with oral squamous cell carcinoma reveals the potentially oncogenic actions of DLEU1. Cell Death & Disease.

[ref-37] Qiu S, Lin S, Hu D, Feng Y, Tan Y, Peng Y (2013). Interactions of miR-323/miR-326/miR-329 and miR-130a/miR-155/miR-210 as prognostic indicators for clinical outcome of glioblastoma patients. Journal of Translational Medicine.

[ref-38] R Core Team (2019). https://www.R-project.org/.

[ref-39] Raulf N, Lucarelli P, Thavaraj S, Brown S, Vicencio JM, Sauter T, Tavassoli M (2018). Annexin A1 regulates EGFR activity and alters EGFR-containing tumour-derived exosomes in head and neck cancers. European Journal of Cancer.

[ref-40] Renaud G, Stenzel U, Maricic T, Wiebe V, Kelso J (2015). deML: robust demultiplexing of Illumina sequences using a likelihood-based approach. Bioinformatics.

[ref-41] Robertson AG, Kim J, Al-Ahmadie H, Bellmunt J, Guo G, Cherniack AD, Hinoue T, Laird PW, Hoadley KA, Akbani R, Castro MAA, Gibb EA, Kanchi RS, Gordenin DA, Shukla SA, Sanchez-Vega F, Hansel DE, Czerniak BA, Reuter VE, Su X, de Sa Carvalho B, Chagas VS, Mungall KL, Sadeghi S, Pedamallu CS, Lu Y, Klimczak LJ, Zhang J, Choo C, Ojesina AI, Bullman S, Leraas KM, Lichtenberg TM, Wu CJ, Schultz N, Getz G, Meyerson M, Mills GB, McConkey DJ, Weinstein JN, Kwiatkowski DJ, Lerner S (2017). Comprehensive Molecular Characterization of Muscle-Invasive Bladder Cancer. Cell.

[ref-42] Robin X, Turck N, Hainard A, Tiberti N, Lisacek F, Sanchez JC, Muller M (2011). pROC: an open-source package for R and S+ to analyze and compare ROC curves. BMC Bioinformatics.

[ref-43] Rothe F, Ignatiadis M, Chaboteaux C, Haibe-Kains B, Kheddoumi N, Majjaj S, Badran B, Fayyad-Kazan H, Desmedt C, Harris AL, Piccart M, Sotiriou C (2011). Global microRNA expression profiling identifies MiR-210 associated with tumor proliferation, invasion and poor clinical outcome in breast cancer. PLOS ONE.

[ref-44] Rouette A, Trofimov A, Haberl D, Boucher G, Lavallee VP, D’Angelo G, Hebert J, Sauvageau G, Lemieux S, Perreault C (2016). Expression of immunoproteasome genes is regulated by cell-intrinsic and -extrinsic factors in human cancers. Scientific Reports.

[ref-45] Shang C, Guo Y, Zhang H, Xue YX (2016). Long noncoding RNA HOTAIR is a prognostic biomarker and inhibits chemosensitivity to doxorubicin in bladder transitional cell carcinoma. Cancer Chemotherapy and Pharmacology.

[ref-46] Siegel R, Ma J, Zou Z, Jemal A (2014). Cancer statistics, 2014. CA: A Cancer Journal for Clinicians.

[ref-47] Siegel RL, Miller KD, Jemal A (2017). Cancer Statistics, 2017. CA: A Cancer Journal for Clinicians.

[ref-48] Siegel R, Naishadham D, Jemal A (2013). Cancer statistics, 2013. CA: A Cancer Journal for Clinicians.

[ref-49] Sobral-Leite M, Wesseling J, Smit VT, Nevanlinna H, van Miltenburg MH, Sanders J, Hofland I, Blows FM, Coulson P, Patrycja G, Schellens JH, Fagerholm R, Heikkila P, Aittomaki K, Blomqvist C, Provenzano E, Ali HR, Figueroa J, Sherman M, Lissowska J, Mannermaa A, Kataja V, Kosma VM, Hartikainen JM, Phillips KA, Couch FJ, Olson JE, Vachon C, Visscher D, Brenner H, Butterbach K, Arndt V, Holleczek B, Hooning MJ, Hollestelle A, Martens JW, van Deurzen CH, Water Bvande, Broeks A, Chang-Claude J, Chenevix-Trench G, Easton DF, Pharoah PD, Garcia-Closas M, De Graauw M, Schmidt MK (2015). Annexin A1 expression in a pooled breast cancer series: association with tumor subtypes and prognosis. BMC Medicine.

[ref-50] Therneau TM (2015). https://CRAN.R-project.org/package=survival.

[ref-51] Volinia S, Galasso M, Sana ME, Wise TF, Palatini J, Huebner K, Croce CM (2012). Breast cancer signatures for invasiveness and prognosis defined by deep sequencing of microRNA. Proceedings of the National Academy of Sciences of the United States of America.

[ref-52] Wang JY, Tai JJ (2009). Robust quantitative trait association tests in the parent–offspring triad design: conditional likelihood-based approaches. Annals of Human Genetics.

[ref-53] Wang L, Shi J, Huang Y, Liu S, Zhang J, Ding H, Yang J, Chen Z (2019). A six-gene prognostic model predicts overall survival in bladder cancer patients. Cancer Cell International.

[ref-54] Wang LL, Sun KX, Wu DD, Xiu YL, Chen X, Chen S, Zong ZH, Sang XB, Liu Y, Zhao Y (2017). DLEU1 contributes to ovarian carcinoma tumourigenesis and development by interacting with miR-490-3p and altering CDK1 expression. Journal of Cellular and Molecular Medicine.

[ref-55] Wang Z, Yang B, Zhang M, Guo W, Wu Z, Wang Y, Jia L, Li S, Xie W, Yang D (2018). lncRNA epigenetic landscape analysis identifies EPIC1 as an oncogenic lncRNA that interacts with MYC and promotes cell-cycle progression in cancer. Cancer Cell.

[ref-56] Xu J, Deng X, Tang M, Li L, Xiao L, Yang L, Zhong J, Bode AM, Dong Z, Tao Y, Cao Y (2013). Tyrosylprotein sulfotransferase-1 and tyrosine sulfation of chemokine receptor 4 are induced by Epstein-Barr virus encoded latent membrane protein 1 and associated with the metastatic potential of human nasopharyngeal carcinoma. PLOS ONE.

[ref-57] Xu F, Zhou W, Cao J, Xu Q, Jiang D, Chen Y (2017). A combination of DNA-peptide probes and liquid chromatography-tandem mass spectrometry (LC-MS/MS): a quasi-targeted proteomics approach for multiplexed MicroRNA quantification. Theranostics.

[ref-58] Yan X, Hu Z, Feng Y, Hu X, Yuan J, Zhao SD, Zhang Y, Yang L, Shan W, He Q, Fan L, Kandalaft LE, Tanyi JL, Li C, Yuan CX, Zhang D, Yuan H, Hua K, Lu Y, Katsaros D, Huang Q, Montone K, Fan Y, Coukos G, Boyd J, Sood AK, Rebbeck T, Mills GB, Dang CV, Zhang L (2015). Comprehensive Genomic Characterization of Long Non-coding RNAs across Human Cancers. Cancer Cell.

[ref-59] Yang L, Taylor J, Eustace A, Irlam JJ, Denley H, Hoskin PJ, Alsner J, Buffa FM, Harris AL, Choudhury A, West CML (2017). A gene signature for selecting benefit from hypoxia modification of radiotherapy for high-risk bladder cancer patients. Clinical Cancer Research.

[ref-60] Yu W, Qian C, Yang L (2018). Racial differences in urinary bladder cancer in the United States. Scientific Reports.

[ref-61] Yu G, Wang LG, Han Y, He QY (2012). clusterProfiler: an R package for comparing biological themes among gene clusters. OMICS.

[ref-62] Zhan Y, Wang LDuL, Jiang X, Zhang S, Li J, Yan K, Duan W, Zhao Y, Wang L, Wang Y, Wang C (2018). Expression signatures of exosomal long non-coding RNAs in urine serve as novel non-invasive biomarkers for diagnosis and recurrence prediction of bladder cancer. Molecular Cancer.

[ref-63] Zhang S, Du L, Wang L, Jiang X, Zhan Y, Li J, Yan K, Duan W, Zhao Y, Wang L, Wang Y, Shi Y, Wang C (2019). Evaluation of serum exosomal LncRNA-based biomarker panel for diagnosis and recurrence prediction of bladder cancer. Journal of Cellular and Molecular Medicine.

[ref-64] Zhang C, Peng L, Zhang Y, Liu Z, Li W, Chen S, Li G (2017). The identification of key genes and pathways in hepatocellular carcinoma by bioinformatics analysis of high-throughput data. Medical Oncology.

[ref-65] Zhao L, Zhao H, Yan H (2018). Gene expression profiling of 1200 pancreatic ductal adenocarcinoma reveals novel subtypes. BMC Cancer.

[ref-66] Zhou M, Guo M, He D, Wang X, Cui Y, Yang H, Hao D, Sun J (2015). A potential signature of eight long non-coding RNAs predicts survival in patients with non-small cell lung cancer. Journal of Translational Medicine.

[ref-67] Zhu R, Yang X, Guo W, Xu XJ, Zhu L (2019). An eight-mRNA signature predicts the prognosis of patients with bladder urothelial carcinoma. PeerJ.

