# Supplementary figures and images for: An RNA-sequencing-based transcriptome for a significantly prognostic novel driver signature identification in bladder urothelial carcinoma
Source: PeerJ. 2020 Jul 21;8:e9422. doi: 10.7717/peerj.9422 (PMC7380276; doi:10.7717/peerj.9422)

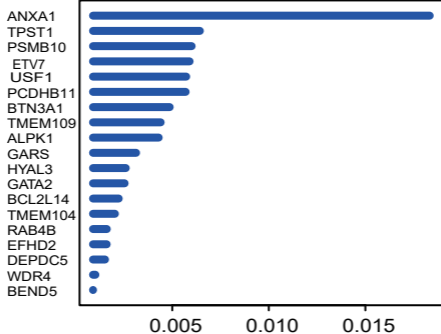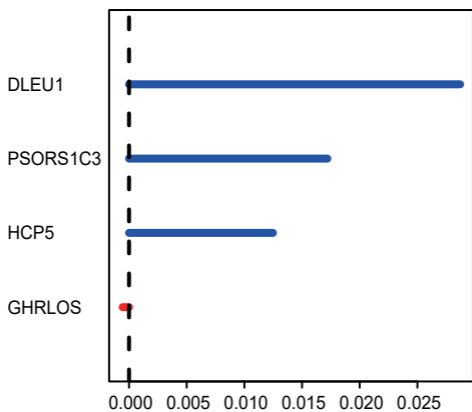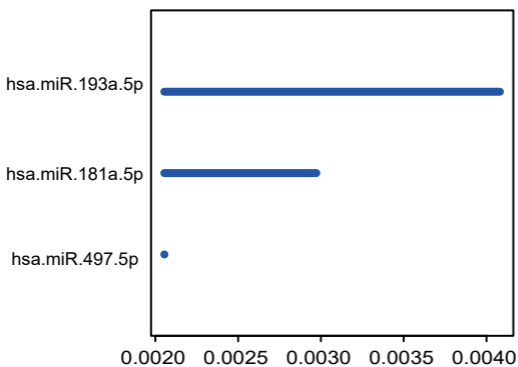

Supplement: Supplemental Information 1 [file peerj-08-9422-s001.pdf]

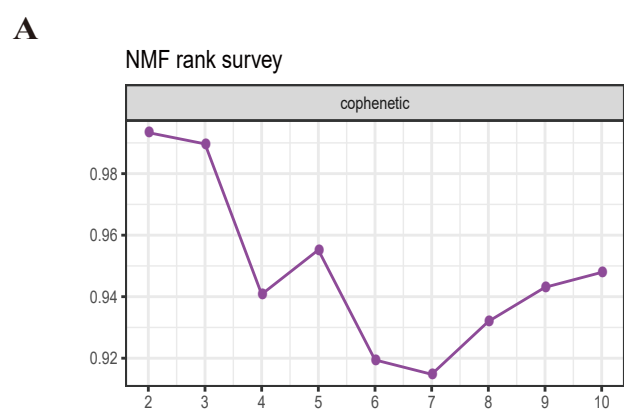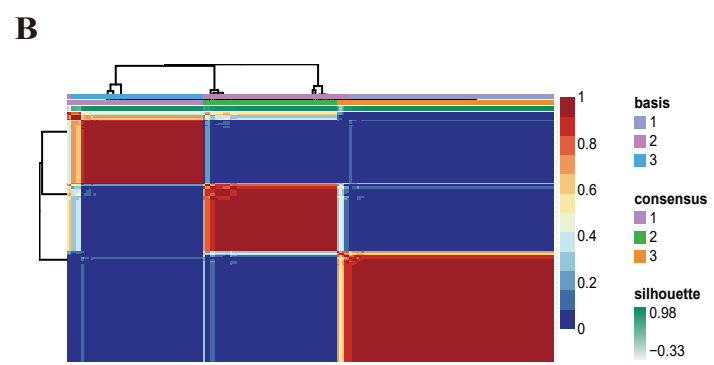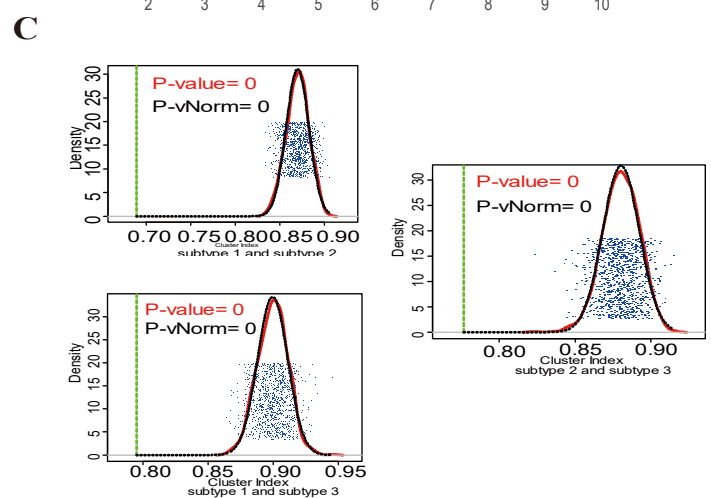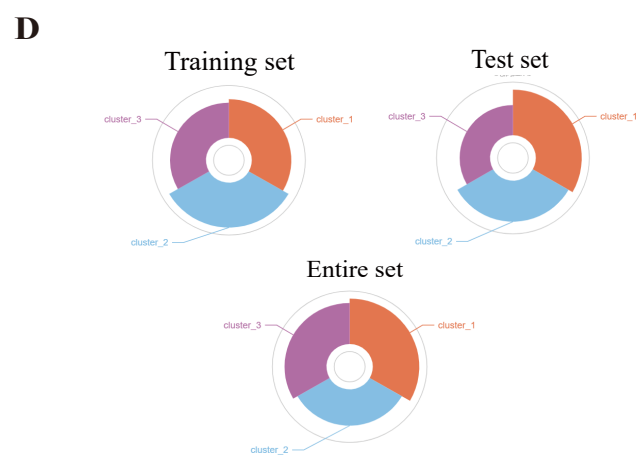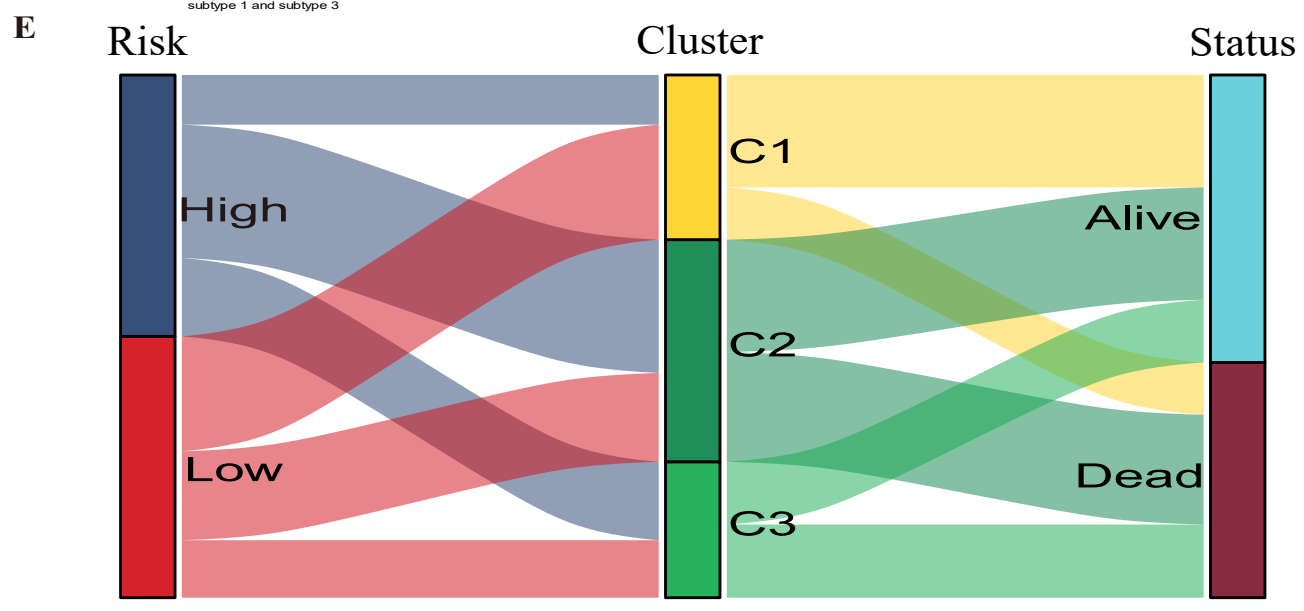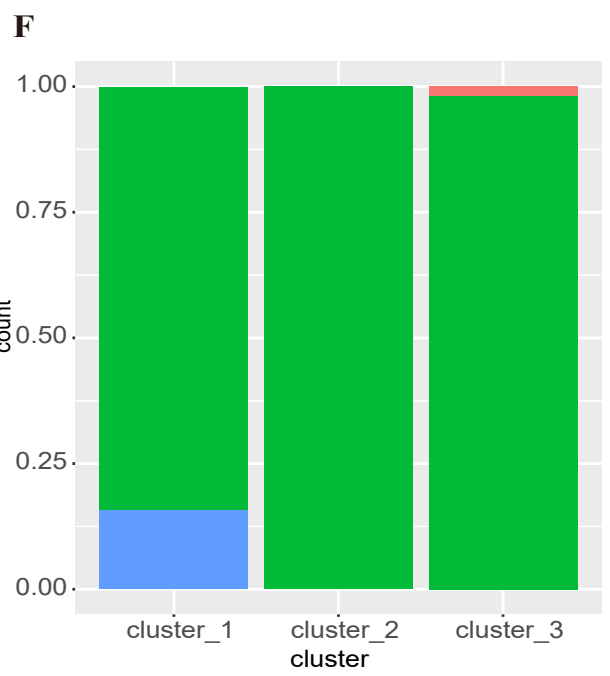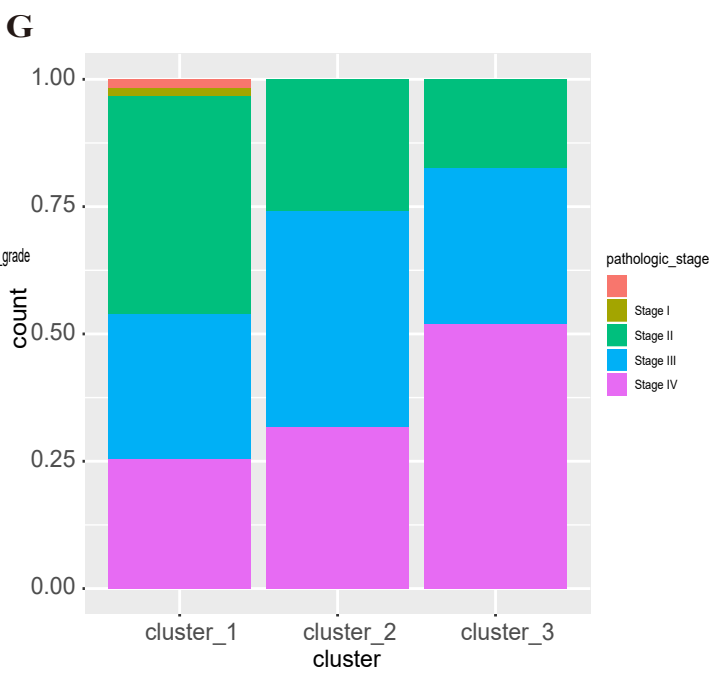

Supplement: Supplemental Information 2 — (A) Unsupervised classification and selection of the appropriate cophenetic correlation coefficient using NMF. A factorization rank of k = 3 was determined as the optimal number of clusters (k). (B) NMF clustering was performed 200 times with optimal k to obtain the NMF consensus matrix. (C) Statistical significance of clustering (SigClust) was performed to validate the significant difference of clustering results in the mRNA expression. (D) Patient distribution into the training set (n = 200), test set ( n = 200) and entire set (n = 400). (E) The correlation of the risk groups, subtypes and clinical status. Distribution of the clinical features, including neoplasm_histologic_grade (F) and pathologic_stage (G) in the identified three clusters. [file peerj-08-9422-s002.pdf]

## Training set

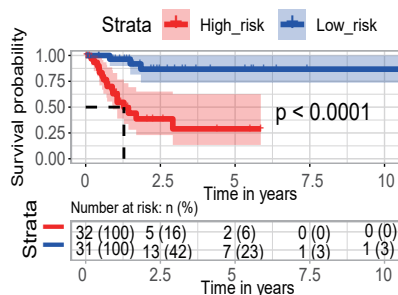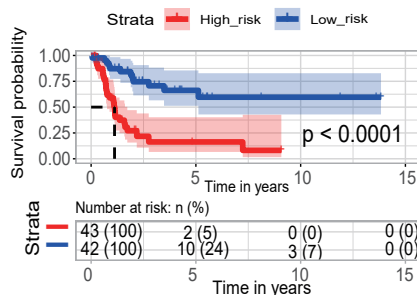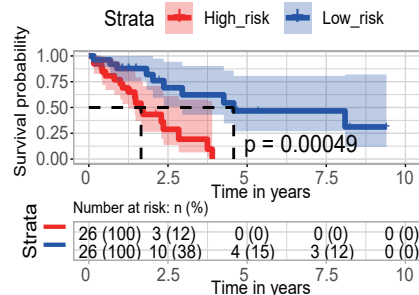

## Test set

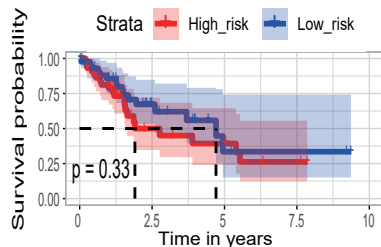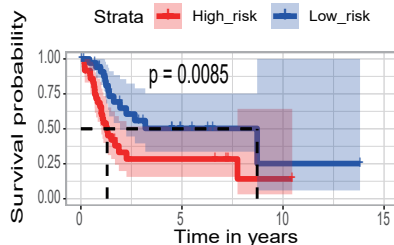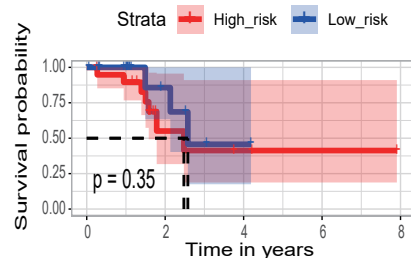

## Entire set

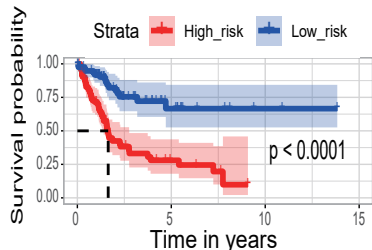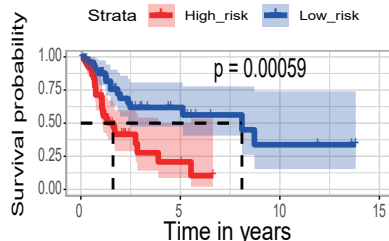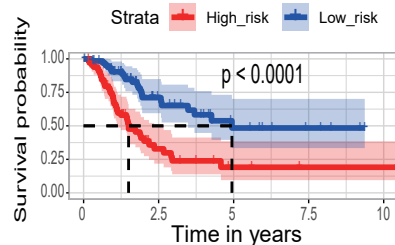

Supplement: Supplemental Information 3 [file peerj-08-9422-s003.pdf]

TCGA\_BLCA 400 samples (n = 400)

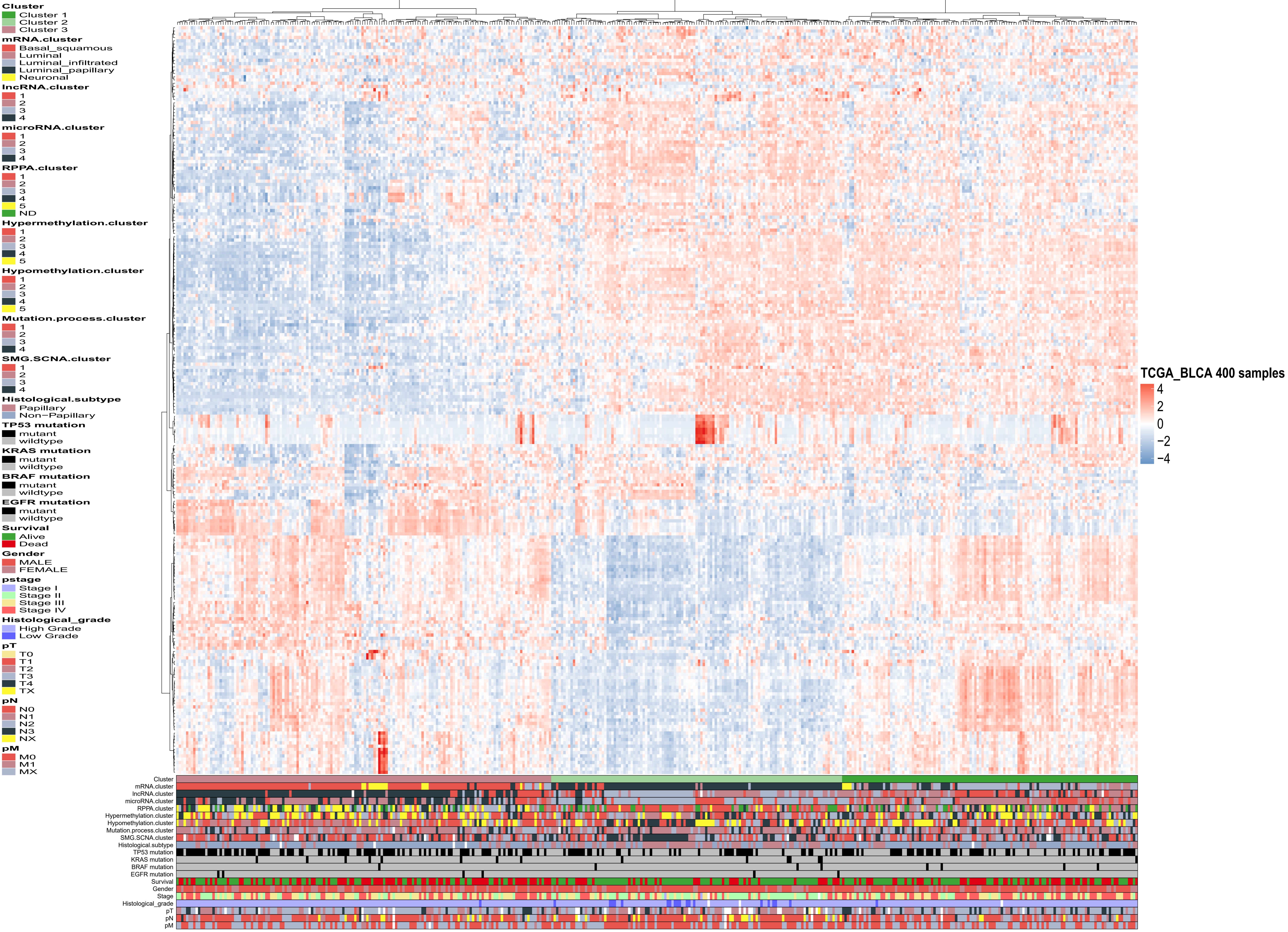

Supplement: Supplemental Information 4 — The heatmap was generated using the heatmap function with SNF algorithm classification, mRNA cluster, lncRNAcluster, microRNA cluster, RPPA cluster, hypermethylation cluster,hypomethylation cluster, mutation process (MSig) cluster, SMG-SCNA cluster, histological subtype, gender, survival status, TNM stages, clinicopathologicalstages, histological grade, and TP53 mutation, KRAS mutation, BRAF mutation, EGFR mutation as the annotations. [file peerj-08-9422-s004.pdf]

**A**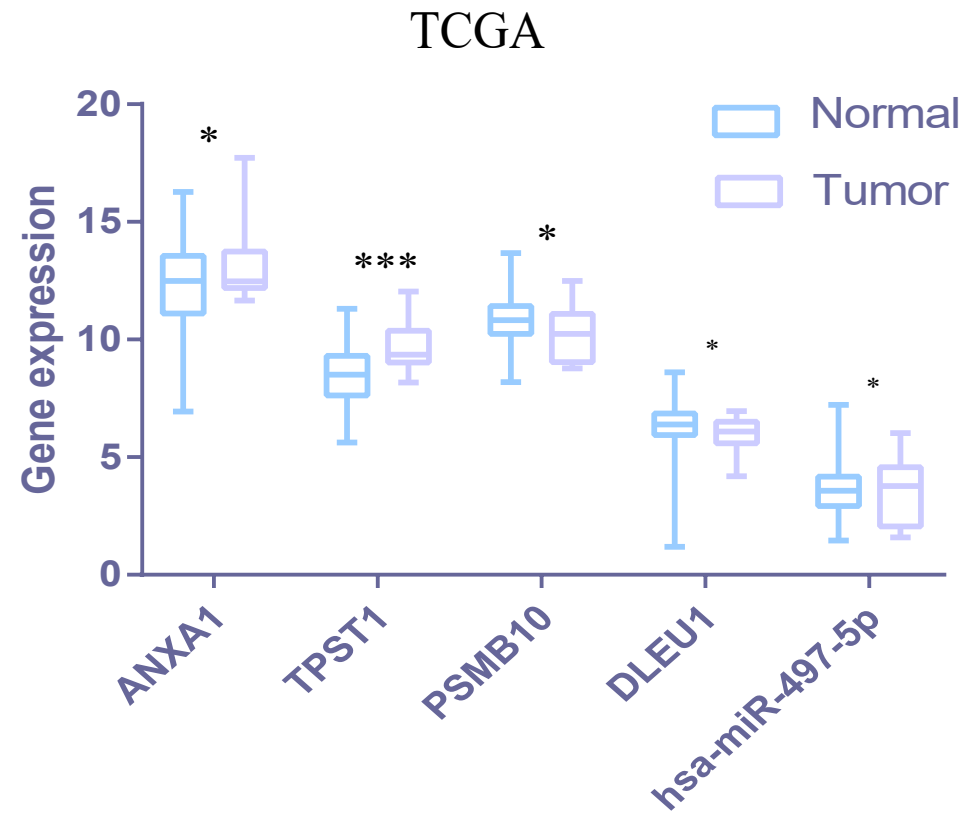**B**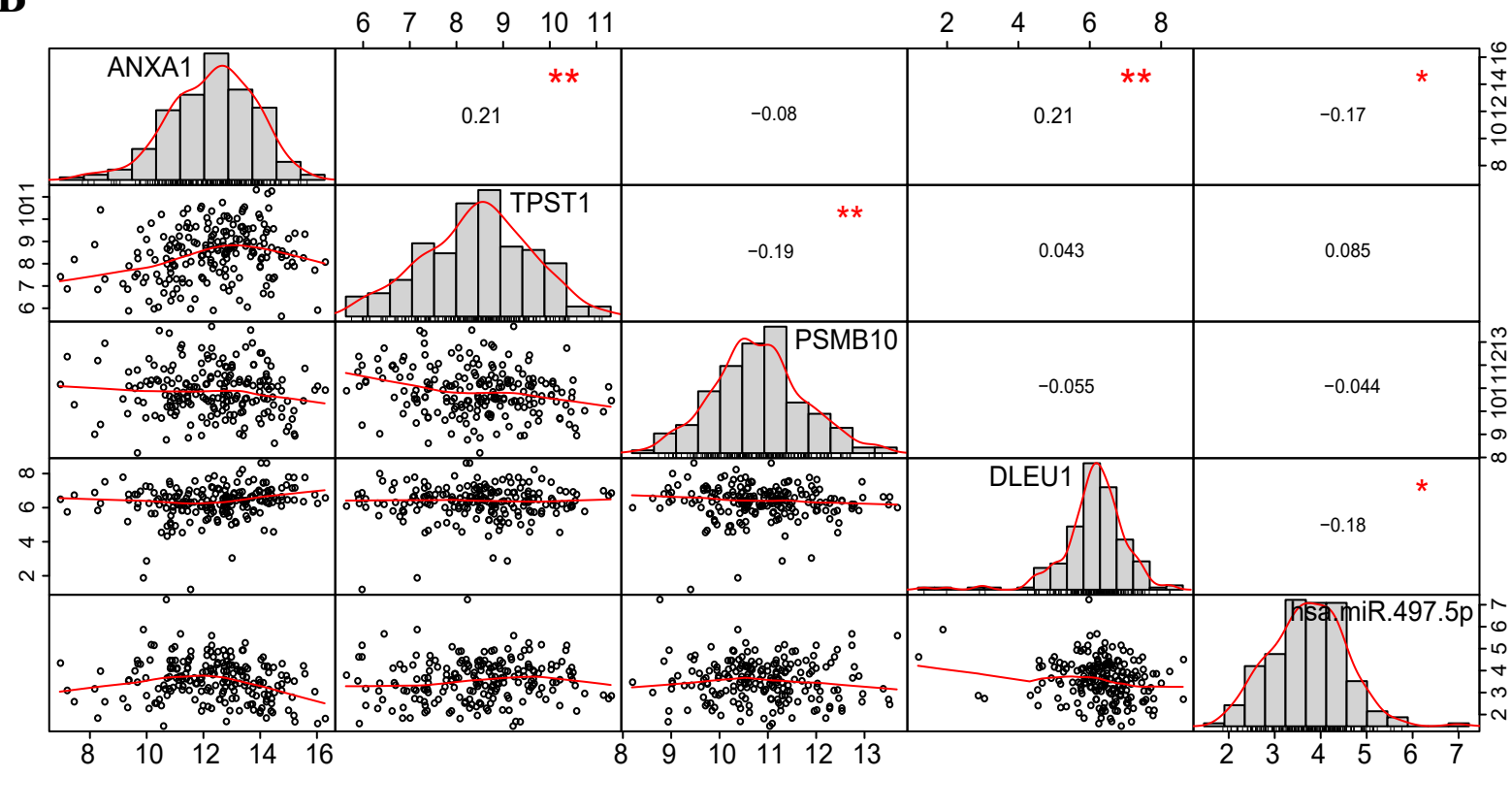

Supplement: Supplemental Information 5 — (A) The basic expression of the five prognostic biomarkers in TCGA. (B) The correlative features of the five prognostic RNAs. The expression correlation analyses of the five feature genes in the whole training set and the subtypes in the training set and normal group. The diagonal is the expression distribution mount of each of the genes; the lower left corner is the gene expression level of the scatter diagram between the two corresponding genes; the upper right corner part is the correlation coefficient of every two genes (ranging from –1 to +1). The significance of the correlation was labelled with “*” (p-value < 0.05) and “**” (p-value < 0.01). [file peerj-08-9422-s005.pdf]
